# The consumer quality index anthroposophic healthcare: a construction and validation study

**DOI:** 10.1186/1472-6963-14-148

**Published:** 2014-04-02

**Authors:** Evi B Koster, Rob RS Ong, Rachel Heybroek, Diana MJ Delnoij, Erik W Baars

**Affiliations:** 1 Professorship Anthroposophic Healthcare, University of Applied Sciences Leiden, Zernikedreef 11, Leiden 2333 CK, The Netherlands; 2 Scientific Centre for Transformation in Care and Welfare (Tranzo), Tilburg University, Tilburg, The Netherlands

**Keywords:** Quality of care, Patient experiences, CQ-index, Anthroposophic healthcare, Validation of measuring instrument, Psychometric quality, Discriminating ability, Patient-centered care

## Abstract

**Background:**

Accounting for the patients’ perspective on quality of care has become increasingly important in the development of Evidence Based Medicine as well as in governmental policies. In the Netherlands the Consumer Quality (CQ) Index has been developed to measure the quality of care from the patients’ perspective in different healthcare sectors in a standardized manner. Although the scientific accountability of anthroposophic healthcare as a form of integrative medicine is growing, patient experiences with anthroposophic healthcare have not been measured systematically. In addition, the specific anthroposophic aspects are not measured by means of existing CQ Indexes. To enable accountability of quality of the anthroposophic healthcare from the patients’ perspective the aim of this study is the construction and validation of a CQ Index for anthroposophic healthcare.

**Method:**

Construction in three phases: Phase 1. Determining anthroposophic quality aspects: literature study and focus groups. Phase 2. Adding new questions and validating the new questionnaire. Research population: random sample from 7910 patients of 22 anthroposophic GPs. Data collection: survey, mixed mode by means of the Dillman method. Measuring instrument: experience questionnaire: CQ Index General Practice (56 items), added with 27 new anthroposophic items added and an item-importance questionnaire (anthroposophic items only). Statistical analyses: Factor analysis, scale construction, internal consistency (Chronbach’s Alpha), inter-item-correlation, discriminative ability (Intra Class Correlation) and inter-factor-correlations. Phase 3. Modulation and selection of new questions based on results. Criteria of retaining items: general: a limited amount of items, statistical: part of a reliable scale and inter-item-correlation <0,7, and theoretical.

**Results:**

Phase 1. 27 anthroposophic items. Phase 2. Two new anthroposophic scales: Scale AntroposophicTreatmentGP: seven items, Alpha=0,832, ICC=4,2 Inter-factor-correlation with existing GP-scales range from r=0,24 (Accessibility) to r=0,56 (TailoredCare). Scale InteractionalStyleGP: five items, Alpha=0,810, ICC=5,8, Inter-factor-correlation with existing GP-scales range from r=0,32 (Accessibility) to r=0,76 (TailoredCare). Inter-factor-correlation between new scales: r=0,50. Phase 3: Adding both scales and four single items. Removing eleven items and reformulating two items.

**Conclusion:**

The CQ Index Anthroposophic Healthcare measures patient experiences with anthroposophic GP’s validly and reliably. Regarding the inter-factor-correlations anthroposophic quality aspects from the patients’ perspective are mostly associated with individually tailored care and patient centeredness.

## Background

The Evidence Based Medicine (EBM) development that started in the nineties of the 20^th^ century has influenced healthcare to a large extent. The main aim of EBM is to improve the quality of healthcare for the individual patient, based on the best evidence available [1].

In association with other developments such as New Public Management from the eighties of the 20^th^ century and subsequently accountability of public finance, there is an increasing demand for scientific and societal accountability of the quality of healthcare [2].

According to the Institute of Medicine [3] quality of care is a multidimensional concept consisting of at least six domains: effectiveness, safety, patient centeredness, timeliness, efficiency and equitably.

In the last two decades one of these domains, patient-centred care, has become increasingly important in relation to the evaluation of the quality of healthcare [4,5]. Patient-centred care represents a humanistic, bio-psychosocial perspective in healthcare, with a strong emphasis on communication, patient participation in clinical decision making, forming a therapeutic alliance and sharing power and responsibility [6].

Various instruments have been developed in order to measure patients’ experiences with patient centeredness, for instance the instruments known as CAHPS (Consumer Assessment of Healthcare Providers and Systems), or the instruments developed by the Picker Institute, and the Consumer Quality Index [7,8].

In 2006, the Dutch Ministry of Health promoted the Consumer Quality (CQ-) Index as *the* instrument to measure patient experiences with healthcare [9,10]. Since then the CQ-Index has been developed in a way that different healthcare services and providers can be measured within the same systematic structure, in order to be able to compare these experiences [8,11].

Currently, several CQ-Indexes have been developed to measure patient experiences with for example: asthma care [12], physiotherapy, hospital specialist care [13], COPD care [14] and general practice [15].

The construction and validation process of a new CQ-index is protocolled by the former Dutch foundation CKZ (*Centre for client experiences in healthcare*). [11] Important parts of this protocol are the formulation of the items and the standardized answer categories, the method of data collection and the statistical analyses.

### Anthroposophic healthcare

Since ideally patient experiences with all areas of healthcare have to be measured, this also is of relevance for anthroposophic healthcare (AH). AH is a form of integrative medicine (IM) that is provided to a small part of the Dutch population (estimation: 200 000 clients) in 80 general practitioner practices [16].

AH emphasizes the relationship between the body, the soul and the mind as well as lifestyle, meaning in life and environmental factors with regard to health and disease. It focuses on the support and active stimulation of physiological self-healing and the self-regulating ability of people. Additional therapies based on the study of anthroposophic humanities, the development and use of natural medicines and a reticent use of conventional chemical medication play an important role within AH. A frequently mentioned and highly appreciated characteristic is the attention to *antropos*, the human being. In practice it means a professional orientation to the individual, an equal relationship between patient and healthcare provider and much time and attention for the patient [16], or in other words: patient-centred care.

AH is strongly organised internationally. This integrative form of healthcare is currently studied and taught at various universities in Europe and ambulatory and clinically practiced in eighty countries worldwide [17].

AH encompasses multiple sectors: general practice, occupational medicine, primary school doctoring, child welfare centres (CWC) clinical specialists, psychiatry, special needs care, and a large amount of paramedic sectors such as physiotherapy, art therapy, eurhythmy therapy and psychotherapy. The GPs work independently and often closely together with these therapeutic disciplines in integrative healthcare centres.

Although the quality and the quantity of the scientific underpinning of AH is growing [17-20], it still suffers from a lack of acknowledgement within conventional healthcare. In the Netherlands as well as internationally large groups of people have good experiences with anthroposophic healthcare [17,21,22].

Nonetheless, up until now, patient experiences with anthroposophic healthcare have not been measured systematically. To enable accountability of quality of AH from the patients’ perspective, the aim of this study is to develop a standardized instrument to measure patients’ experiences with AH objectively and systematically.

This instrument will generate feedback information about patient experiences, which can be used to monitor and improve the quality of AH. The instrument will meet the increased demand for quality assurance of (anthroposophic) healthcare in general and it will contribute to the scientific underpinning and accountability of AH.

The present study focuses on investigating specific anthroposophic aspects of quality of care; the reliability, factorial structure and validity of the CQ-Index Anthroposophic Healthcare; the experiences with these anthroposophic aspects of healthcare in a group of patients at anthroposophic GP practices; and designing a final version of the CQ-Index questionnaire. The development of the CQ-index AH combined the development of a measuring instrument for GP care with a measuring instrument for a broader spectrum of AH therapies. This article focuses on the development of the CQ-index AH for GP care only.

The overall research question was: How can quality of anthroposophic healthcare be measured from the patients’ perspective? More specifically: Which aspects of quality of care are important from the perspective of patients of anthroposophic GP’s? How can these aspects of care be measured as individual items in a valid and reliable way? Which items are part of a valid coherent scale and should be retained? What are patients’ experiences of these aspects of care?

## Methods

The CQ-Index systematic consists of an experience questionnaire and an item-importance questionnaire [11]. According to the CKZ-guidelines the construction of the CQI-AH consisted of three phases. Phase one concerned the determination of the aspects of quality of care that are important from the perspective of patients of anthroposophic GP’s and the construction of ‘anthroposophic questions’. Phase two concerned the adding of new questions to the existing CQ-Index General Practice [15], conducting a survey involving a sample of nearly 8000 patients of the population of anthroposophic GPs and validating the new questionnaire. Phase three concerned the modulating and selection of new questions based on the results in the previous phases.

### Ethics statement

Regarding research ethics it is important to mention under Dutch legislation, which is laid down in the *Medical Research Involving Human Subjects Act*[23]*,* this study is not subject to approval of an ethics commission. The *Declaration of Helsinki* does not apply on this study, because it is not medical research, but social science. The patients’ experiences are used to evaluate the performance and service of doctors. There is no medical data involved.

However, the study is carried out according to protocol of the former foundation CKZ (Centre for patient experiences in healthcare) [24]*.* The protocol is written down in the *Handbook CQI development*[11]. All necessary research ethics are covered within this protocol and the protocol was tested on it. The protocol is also fully in line with the legislation of the Dutch DPA (Data Protection Authority). The researchers followed this protocol in detail.

### Phase 1: determining anthroposophic aspects

In order to determine what the anthroposophic aspects of GP care are, the CKZ-guidelines prescribe literature review and focus groups and/or interviews with experts. Rather than having both focus groups and interviews it was chosen to invite all participants in the focus group process. In this way patients and professionals could actually have a discussion on quality aspects together.

The literature was reviewed and three focus groups were organised.

#### Literature study

For the literature study a database search of Pubmed, Google Scholar, the database of the international anthroposophic Medical Section [25] and the internet in general was executed. The search terms were (a combination of): ‘quality of care’ , ‘anthroposophy’ , ‘anthroposophic healthcare’ ‘patients’ perspective’. In addition, the Dutch anthroposophic occupational associations and the Dutch anthroposophic healthcare institutions were asked for client questionnaires with specific anthroposophic items. Finally, the Dutch patient organisation for AH, Antroposana, was consulted for literature on the subject of quality aspects of anthroposophic care from the perspective of their members.

#### Focus groups

Because of the wider context of the study regarding the broad spectrum of anthroposophic therapies both anthroposophic GP care and anthroposophic therapist care were discussed in the focus groups. Regarding the composition of the focus groups the guidelines of the CKZ were followed. A minimum of 6 participants was compulsory.

In the first focus group (with three patients, three healthcare professionals, and one chairman) specific domains of anthroposophic experiences were determined and corresponding conceptual questions were formulated. The second focus group was an enlargement of the first focus group. A broad spectrum of anthroposophic therapists (13), doctors (4) and several patients (8) gave their feedback on the conceptual domains and questions that were constructed as a result of the first focus group. To minimalize group- and information management issues the communication in the second focus group went through email. In the third focus group (with two patients, two healthcare professionals, and two researchers) the feedback from the second focus group was discussed. All the professional views were in the end discussed with the patients’ representations.

The patients were recruited from the Dutch patient organisation for AH, Antroposana. In total nine different patients were consulted for the construction of the questionnaire, two of them were present at all focus groups. The doctors and therapists were recruited from their anthroposophic occupational associations. All healthcare professionals had a working experience of a minimum of 5 years. In the consensus process with the members of the focus group and the anthroposophic patient organisation additional items were formulated. After that they were tested cognitively. The questionnaire was sent to 13 members of the anthroposophic patient organisation. They were asked if the questions and the structure of the questionnaire were understandable. After integrating the criticisms the questions were added to the existing CQ-Index General Practice [15] and a new questionnaire was constructed.

### Phase 2: validation of the CQ-index AH

#### Research population

The new questionnaire was tested in a survey involving a patient population among 22 Dutch anthroposophic GPs. (Total population: 29 341 patients) Following the guidelines of the CKZ [11] two random samples were taken. One random sample consisted of 6910 patients of 22 anthroposophic GP practices (range: 250-475 per practice).

According to the guidelines 20 practices were needed. It was chosen to include two spare practices.

Following the recommendations of the pilot study CQ-Index GP [15] only patients aged 18 years and older who visited their GP in the last year were included. They received the newly constructed questionnaire called the experience questionnaire. This questionnaire was to be validated. After the first sample a smaller random sample of 1000 patients (50 per practice) was taken from 20 of the original 22 anthroposophic GP practices. In this sample no spare practices were needed. These patients received the item-importance questionnaire. This questionnaire resulted in an importance rating per item. This rating was part of the criteria for the item inclusion in the factor-analyses of the experience questionnaire. It was chosen to send both questionnaires to different samples in order not to burden the respondents with more than one questionnaire.

The CKZ-guidelines describe that the samples need to be representative in comparison to the population. This is checked for each GP practice individually while taking the samples.

#### Data collection

The data collection was primarily accomplished online. This was also a recommendation of the pilot study CQ-Index GP [15], in order to be able to cut costs and save paper. Respondents received an invitation by mail from their GP practice with an Internet link and a login. A supplement in English, Turkish and Arabic was attached. People who didn’t want or had difficulties responding online could ask for a paper version of the questionnaire.

Two weeks after the first invitation the whole sample received a thank-you/reminder postcard and four weeks after the first invitation only the non-responders received a reminder invitation containing the Internet link and the login again. The data collection took place in accordance with the privacy legislation of the Dutch DPA (Data Protection Authority).

#### Measuring instruments

The experience questionnaire measured patients’ experiences with quality of care. The questionnaire contained 83 items, of which 56 from the existing *CQ-Index GP* and 27 new anthroposophic items. The response options of the majority (69) of the items were*: never (1), sometimes (2)*, *usually (3)* and *always (4).* Six items had response options *yes* and *no*. One item was a global rating of the GP practice, with response options from 0 (worst possible) to 10 (best possible) and one item was a net promotor score. Seven items had other response options and two items had open answers.

The item-importance questionnaire contained the same questions, only reformulated to ask about patients’ opinions of the importance of the 27 new anthroposophic questions.

The answer categories were: *not important (1), quite important (2), important (3) and very important (4)*. Finally, both questionnaires contained questions regarding the background characteristics of the respondent.

#### Statistical analyses

Univariate and multivariate statistical analyses were conducted in order to test the validity of the new items and gather information needed to construct a new CQ-Index AH.

Three consecutive factor analyses were performed to explore if the new items led to new factors: a first factor analysis to establish the factorial structure of the new items, a second factor analysis to determine if these factors contributed to the factorial structure of the original questionnaire and the last factor analysis to test if additional anthroposophic questions loaded on original factors. After the construction the new scales were checked to confirm that each of them didn’t consist of more than one factor.

The items were analysed individually before including them in the factor analyses. The criteria for inclusion of new items in the factor analyses were: comparable answer-structure, number of missing values (combined with the answers ‘don’t know’ and ‘not applicable’), importance rating, inter-correlation of the answers of separate questions. The discriminative ability was determined by means of intra-class-correlation (ICC) scores of the new scales and items.

Subsequently, the statistical quality of new scales (and items) was tested by determining the intra-class-correlation (ICC), factor loadings, item-total-correlation (ITC) and Crohnbach’s alpha if item deleted. Also, inter-factor-correlations between both new and original scales were calculated.

### Phase 3: modelling and selection of new items

In order to decide which items were to be enclosed in the final measurement instrument, general, statistical and theoretical criteria were followed. The general criterion was to only add a limited number of necessary items contributing to new reliable scales with additional value. The statistical criteria were that items were part of a reliable scale and had inter-item-correlation <0,7. The theoretical criteria concerned the content of AH. The final decision per item was made in dialogue with the Dutch anthroposophic GP’s occupational association and the Dutch anthroposophic patient organisation.

## Results

### Phase 1: anthroposophic quality aspects

#### Literature study

The Dutch patient organisation for AH formulated their quality criteria of AH in 9 domains: Autonomy, Treatment attitude, Support, Therapy, Expertise, Information, Accommodation, Organisation, and Professional regulation [26].

The following validated questionnaires are used: CQ-Index Huisartsenzorg Overdag [27], CQ-Index Kortdurende ambulante GGZ [28], Vragenlijst levenshouding (SOC scale) [29], Levensvisie vragenlijst [30], Werkalliantie vragenlijst [31], IDQOL-16 [32] and Vragenlijst Jeugdthermometer [33]. Three non-validated questionnaires from three healthcare centres were also used.

#### Focus groups

Specific aspects that are associated with anthroposophic care mentioned in the focus groups are (amongst others): Being heard as a human being; individually tailored care; attention to physiological self-healing; autonomy of the patient; deeper insight in one own health problem; less side effects; body, soul and mind as a unity; conscious integration of spirituality or view on life within treatment; interdisciplinary cooperation; enough time for the patient and possibility of choosing for conventional and/or anthroposophic treatment.

In the focus groups the following domains of AH have been formulated: Practice/organisation, Cooperation between healthcare professionals, Life vision and spirituality, Therapeutic relationship, Physiological self-healing, self-management, Individually tailored care, Expertise and insight, Structure of treatment and Healing Environment.

These domains led to the formulation of 27 additional items. These items were added to the existing experiences questionnaire CQ-index GP and reformulated into an item-importance questionnaire (only AH items).

### Phase 2: validation of the CQ-index AH

#### Response rate

The experience questionnaire had a gross response rate of 35, 4% and a net response rate of 30, 6% (N_GP_ = 2063) (Table 1).

**Table 1:** Response analysis

		**N**_**GP**_	**% of net sample**	**% of gross response**	**N**_**IM**_	**% of net sample**
**Gross sample**		6910			1000	
	Not registered at GP	100			20	
	No visit in last 12 months	75			-	
	Younger than 18	2			1	
**Net sample**		6733	100%		979	100%
	Non response with notification	23			9	
	Recently died	4			-	
	Non response without notification	4322			625	
**Gross response**		2384	35,4%	100%	345	35,2%
	Not completed questionnaires (digital)	281			26	
	Less than 50% of compulsory questions	2			-	
	Paper and digital	2			-	
	Only therapist questionnaire	36			-	
	Other	-			4	
**Net response**		2063	30,6%	86,4%	315	32,2%

The item-importance questionnaire had a response rate of respectively 35, 2% and 32, 2% (N_IM_ = 315). (Table 1) 65% of the responders were female and 35% were male. The mean age of the responders was 54 years (sd = 15) (Table 2).

**Table 2:** Sample and response: age and gender

	**Net sample**	**Net response**
**Experience**	6733	2063
% male	*38,9*	*34,8*
% female	*61,1*	*65,2*
Age, mean	49,36	54,53
(SD)	(17,45)	(15,40)
% under 45 years	*40,8*	*25,1*
% 45 years and above	*59,2*	*74,9*
**Item-importance**	979	315
% male	*38,1*	*35,9*
% female	*61,9*	*64,1*
Age, mean	49,27	54,01
(SD)	(17,08)	(14,63)
% under 45 years	*39,1*	*24,4*
% 45 years and above	*60,9*	*75,6*

The responders are on average high educated. More than 55% had a HBO ^a^[1] or university education. 78% considered his or her general health good to excellent. 14% had used the possibility to answer the questionnaire on paper instead of online. In comparison with the sample, women responded more often (experience: p < 0,000, item-importance: not significant) as well as people aged >45 years (experience and item-importance: p < 0,000).

#### Item analysis

There were 4 items with high percentages (>50%) missing and no items with extreme skewness. 8 items had a high inter-correlation (r > 0, 7) with one or more other items. Table 3 shows details about the item analyses. Table 4 shows the importance rating per item. There were 11 items with a high importance rating (>3) (Table 3 and 4)

**Table 3:** Item analyses, factor analyses and item selection regarding new anthroposophic scales

	**Don’t know (%)**	**Not applicable (%)**	**Total missings (%)**	**High inter-item correlation [item]**	**Importance rating >3,0:+ or <2,5:-**	**Including in factor analyses: + or -**	**Factor loading (factor) analysis 1**	**Item-total correlation**	**Additional theoretical reason for including or excluding**
**Practice**									
22. Ervoer u de inrichting van de wacht- spreek/behandelkamer als positief op uw welzijn?	10,1		12,2		-	-			Ex:-
*(inside environment of practice building)*									
**GP’s interactional style**									
43. Stelde uw huisarts u op uw gemak?			2,2		+	+	0,746	0,711	In:-
*(making comfortable)*							(1)		
44. Had uw huisarts voldoende begrip voor uw klacht of aandoening?			2,1		+	+	0,782	0,764	In:-
*(showing understanding)*							(1)		
45. Gaf uw huisarts u inzicht over de achtergrond en mogelijke oorzaken van uw klacht of aandoening?			2,5	[46]	+	+	0,743	0,724	In:-
*(providing insight in background and causes)*							(1)		
46. Hielp uw huisarts u omgaan met de ongemakken en beperkingen die de klacht/aandoening met zich meebrengt?	6,8	50,5	59,2	[45,47]		+	0,718	0,766	Ex: item- reduction, inter-item correlation
*(helping to cope with constraints)*							(1)		
47. Had uw huisarts aandacht voor de eventuele gevolgen van uw klacht/aandoening voor uw sociale leven?			3,5	[46]		+	0,756	0,777	Ex: item- reduction, inter-item correlation
*(attention for social consequences)*							(1)		
48. Bood uw huisarts u de zorg die u op dat moment nodig had?			2,3		+	+	0,783	0,776	In:-
*(providing needed care)*							(1)		
49. Had uw huisarts een goede balans tussen betrokkenheid en professionele afstand?	10,4		12,5		+	+	0,690	0,611	In:-
*(balanced attitude)*							(1)		
**Treatment/intervention**									
68. Bent u door uw huisarts goed geïnformeerd over de antroposofische behandelingsmogelijkheden?			4,0			+	0,421	0,548	In:-
*(anthroposophic treatment options)*							(2)		
69. Heeft uw huisarts u vrij gelaten in de keuze voor een reguliere en/of antroposofische behandeling?		25,5	28,9	[70]	+	+	0,844	0,626	In: Medical–ethical value
*(free choice of treatment options)*							(3)		
70. Heeft uw huisarts u vrij gelaten in de keuze voor chemische of natuurlijke medicatie?		21,2	24,6	[69]	+	+	0,863	0,620	Ex:-
*(free choice of type of medication)*							(3)		
71. Waren u en uw huisarts het eens over hoe uw klacht of aandoening te behandelen?		9,7	12,7		+	+	0,611	0,631	Ex: Item reduction, partly covered by existing item 31
*(consensus about treatment)*							(1)		
73. Richtte uw huisarts de behandeling (ook) op het ondersteunen van de eigen herstelkrachten van uw lichaam?	24,6		27,8		+	+	0,625	0,655	In:-
*(focus on physiological self-healing)*							(2)		
**Anthroposophic view**									
74. Kon u door de antroposofische benadering beter begrijpen wat er met u aan de hand was?	27,6		31,2	[78]		-			Ex:-
*(provinding insight in situation)*									
75. Werd u door uw huisarts aangesproken in uw eigen verantwoordelijkheid voor de keuzes die u maakt met betrekking tot uw gezondheid?	15,7		19,4		+	+	0,749	0,586	In:-
*(patients’ own responsibility)*							(2)		
76. Motiveerde de aanvullende benadering u om zelf actief aan uw gezondheidstoestand bij te dragen?	17,6		21,3		+	+	0,829	0,706	In:-
*(patients’ active contribution)*							(2)		
77. Ondersteunde de arts u hierbij op voor u belangrijke momenten?	5,7	39,7	48,6			+	0,729	0,710	Ex: item reduction, combination with 76
*(support of GP)*							(2)		
78. Kon u door de antroposofische benadering uw klacht/aandoening betekenis geven binnen de samenhang van uw leven en functioneren?	11,2	36,8	51,2	[74,79]		-			In: theoretical importance content AH
*(giving meaning with daily life)*									
79. Ondersteunde de antroposofische benadering u in uw persoonlijke ontwikkeling als mens?	11,0	38,4	52,3	[78]		-			Ex:-
*(support on personal growth)*									
80. Bood de antroposofische benadering u handvatten (of inspiratie) om uw klachten/aandoening een spirituele betekenis te geven?	10,5	40,5	54,1		-	-			Ex:-
*(spiritual meaning)*									
**Perceived treatment effect**									
83. Waren er bijwerkingen van de behandeling?	21,4		25,7			+	0,436	0,141	In: theoretical and scientific importance: separate item.
*(prevalence of side effects)*							(3)		
84.Beïnvloedde de behandeling de kwaliteit van uw leven op een positieve manier?	24,0		28,2		8	+	0,663	0,567	In:-
*(influence on quality of life)*							(1)		
**Separate items with other answer catagories**									
***(no factor analyses)***									
67. Wat is de belangrijkste klacht of aandoening waarvoor u de afgelopen 12 maanden in behandeling bent geweest?									Ex: item reduction, not compulsory for respondent
*(main complaint/illness, open item)*									
72. Hoe bent u behandeld?			16,2						In: comparison between AH and conventional care
*(way of treatment)*									
81. Heeft de antroposofische benadering u verwachtingen gegeven ten aanzien van het verbeteren van uw gezondheid?		34,7	3,3						In: scientific importance
*(expectations of AH treatment)*									
82. Binnen welke termijn heeft u positief effect van de behandeling ervaren?		9,7	5,1						Ex: joined with 67
85. Zo ja, kunt u aangeven waarom?									In: joined with 84
*(If so, please explain why? open item)*									

**Table 4:** Importance ratings

**Items**	**Importance rating**
**Practice**	
22. *(inside environment of practice building)*	2,5
**GP’s interactional style**	
43. *(making comfortable)*	3,1
44. *(showing understanding)*	3,4
45. *(providing insight in background and causes)*	3,4
46. *(helping to cope with constraints)*	3,0
47. *(attention for social consequences)*	2,8
48. *(providing needed care)*	3,4
49. *(balanced attitude)*	3,2
**Treatment/intervention**	
68. *(anthroposophic treatment options)*	2,9
69. *(free choice of treatment options)*	3,1
70. *(free choice of type of medication)*	3.1
71. *(consensus about treatment)*	3,1
73. *(focus on physiological self-healing)*	3.2
**Anthroposophic view**	
74. *(providing insight in situation)*	3,0
75. *(patients’ own responsibility)*	3,0
76. *(patients’ active contribution)*	3,1
77. *(support of GP)*	2,9
78. *(giving meaning within daily life)*	2,9
79. *(support on personal growth)*	2,7
80. *(spiritual meaning)*	2,1
**Perceived treatment effect**	
83. *(prevalence of side effects)*	2,7
84. *(influence on quality of life)*	3,1
**Separate items with other answer catagories: *no importance analysis***	**-**

#### Factor analyses

The first factor analysis showed a structure of three components, (component one: items 43-49,71,77, component two: items 68,73,75-77,84 and component three: items 68-70,83). The second factor analysis showed one component with exclusively additional items (68,73,75-77,84). The third factor analysis showed the impact of the additional items on the old scales. Two existing scales (Accessibility and Reception) didn’t change. One additional item (43) loaded on the scale SocialHandlingGP. 10 items (43-49, 68, 71 and 77) loaded on the scale CommunicationGP, and 8 items (43-49 and 71) loaded on the scale TayloredCareGP. Also, the scale TayloredCareGP divided into three components. A fourth factor analysis confirmed the newly constructed scales and showed only minor shifts in factor loadings.

#### Scale construction

Finally, two new scales regarding AH were constructed. The first scale contains items about the treatment attitude of the GP and the therapeutic relationship between the GP and the patient. This scale is called InteractionalStyleGP. The second scale contains items concerning the content of the anthroposophic approach within the treatment from the GP. This scale is called AnthroposophicTreatment. Table 3 shows details about the item analysis and factor analyses that were the basis of the item selection regarding the new scales and Table 5 shows the final item content of the two scales

**Table 5:** New scales

**Scale**	**Items**	**Mean scale score**	**Conbach’s alpha**
**InteractionalStyleGP**	43. Making comfortable	3.6	0.810
	44. Showing understanding		
	45. Providing insight in background and causes		
	48. Providing needed care		
	49. Balanced attitude		
**AnthroposophicTreatment**	68. AH treatment options	3.1	0.832
	69. Free choice of treatment options		
	73. Focus on physiological self-healing		
	75. Patients’ own responsibility		
	76. Patients’ active contribution		
	78. Giving meaning within daily life		
	84. Influence on quality of life		

#### Psychometric quality

Both scales are sufficiently internally consistent (Cronbach’s Alpha > 0, 7) (Table 5).

The correlations between the existing and the new scales vary strongly (Table 6). Both new scales have their highest correlation with the existing TailoredCareGP scale (AnthroposophicTreatment: r = 0, 56; InteractionalStyleGP: r = 0, 76). The correlation between the two new GP scales is r = 0, 50. The AnthroposophicTreatment scale has low to moderate correlations with all other scales.

**Table 6:** Pearson’s correlations between existing and new anthroposophic scales

	**Interactional style GP**	**Anthroposophic treatment**
Existing scales GP		
**Accessibility**	.32*	.24*
**Reception**	.38*	.26*
**SocialHandlingGP**	.70*	.36*
**CommunicationGP**	.75*	.41*
**TailoredCareGP**	.76*	.56*
**InteractionalStyleGP**	1	.50*

* p < 0.05.

The ICC values (InteractionalStyleGP: ICC=5,7; AnthroposophicTreatment: ICC=4,2) demonstrate that these scales are able to determine small though statistically significant differences between the GP practices.

#### CQ-index AH scores

The mean overall value for patients’ global rating of the GP practices is 8,4 (range 0-10; sd=1,2).

The mean scale scores, following the answer categories, (range 1(never) - 4(always)) are: InteractionalStyleGP: 3,6; AnthroposophicTreatment: 3,1 (Table 5). The mean scale scores equal the answer categories *usually* and *always.*

The mean overall value and the mean scale scores demonstrate an overall positive image of the anthroposophic GP care.

### Phase 3: final questionnaire

The final questionnaire consists of the complete CQ-Index GP added with two anthroposophic scales. The scale InteractionalStyleGP contains five items. The scale AnthroposophicTreatment contains seven items. Four items remain singular items because of their importance for AH. The complete final questionnaire is provided in an additional file. (Additional file 1)

## Discussion

In this study a CQ-index AH has been constructed and tested among 2063 patients in 22 GP practices. The CQ-index AH contains the complete CQ-Index General Practice, two new anthroposophic scales and four singular items. The internal consistency of the two new scales is sufficient and the discriminating ability of the two new GP scales is small though statistically significant.

The results of the measurement of client experiences in AH practices are positive. The mean scale scores demonstrate positive experiences with anthroposophic care. The anthroposophic GP practices are valued with a mean of 8,4 on a scale from 0 to 10.

The recommendation to invite people to an online questionnaire has been successfully brought in practice: 87% of the responders used the Internet. The general response was lower than expected, though the difference between the gross and the net response was small. Due to the digital data collection most of the questionnaires (86, 4%) could be used for analysis.

It seems obvious to think that the relatively low response rate was related to the comprehension of the added questions and the length of the questionnaire. However, this does not show up from the number of people who started, but did not finish the questionnaire. Also, there is hardly any difference in response percentage between the relatively long experiences questionnaire and the relatively short item-importance questionnaire.

The most explicit characteristics of the responders are the high percentage of higher education and the majority being female and over 45 years of age. These characteristics differ only slightly from the international research [17] about the socio-demographics of the users of AH. Kienle [17] demonstrates that anthroposophic treatment is used particularly by well-educated women aged between 30 and 50 years.

The adding of the two new scales demonstrates that it is possible to measure patients’ experiences with stimulation of physiological self-healing, the active contribution to one’s own health, the doctor-patient relationship, and the interactional style of the healthcare professional.

Previous research on the Consumer Quality Index has demonstrated the importance of the doctor-patient relationship and communication. Patients’ global rating of their healthcare is strongly determined by their experiences with interpersonal aspects of care and doctor-patient communication [34]. By adding these aspects the CQ-Index AH enhances the CQ-Index structure and extends the theory of client experiences with healthcare in general.

The factor analyses demonstrate that the items regarding self-management in combination with other anthroposophic treatment items make a good independent scale. The correlation of this scale (AnthroposophicTreatment) with all other GP-scales is small to moderate. The highest correlation of both new scales is found with the scale TayloredCare (AnthroposophicTreatment: r = 0, 56; p < 0, 05 and InteractionalStyleGP r = 0, 76; p < 0, 05). There is also a high correlation between InteractionalStyleGP and resp. SocialHandlingGP (r = 0, 70; p < 0, 05) and CommunicationGP (r = 0, 75; p < 0, 05).

These correlations demonstrate that the anthroposophic aspects of care are relatively strongly related to individually tailored care and a social handling focussed on having an alliance with the individual human being. Patients especially value this treatment attitude or interactional style. This is an important reason why the scale InteractionalStyleGP is maintained, despite its high correlation with these three scales. Further research needs to be done among a patient population of both anthroposophic and conventional GP’s to determine if and how these scales can be integrated in order to reduce the amount of items.

The rating of the anthroposophic GPs is slightly higher than the mean rating of the conventional GPs (8, 2) measured in 2008 [15] and 0, 5 point higher than the mean rating of GPs in 2010 (7, 9) [13]. To illustrate: other mean ratings are (for example): Asthma care: 7, 0 [12] Physiotherapist: 8, 0, Hospital specialist: 7, 7 [13] and COPD care: 7, 7 [14]

Since the anthroposophic aspects are part of a broader approach to practicing healthcare, emerging in the current developments of self-management, tailored care and health promotion it would be interesting to measure the anthroposophic items in conventional GP practices.

### Limitations

One of the limitations of this study regards the general response. Compared to the pilot study in 2008 the response was again lower than expected. The recommendations from this previous study to invite only the people who have been to their GP in the last year and only people from 18 years and older have not led to a higher response.

Another limitation of this study is the discriminating ability of the instrument. The small though statistically significant discriminating ability enables this instrument of measuring differences between practices, although possibly large samples are needed. Supplemented research about the size of these samples is recommended here. Its discriminating ability might be higher and more relevant if tested among conventional and anthroposophic practices together. However, the expectations should not be too high, because the discriminative ability of patient experience surveys is sometimes rather limited [35].

### Future perspectives

With the development of the CQ-Index AH there is a standardized and validated instrument to measure patient experiences within the AH systematically and take account of the quality of AH from the patients’ perspective. The future perspectives of this instrument are the possibility of comparing anthroposophic institutes and groups of professionals, meeting future criteria regarding monitoring and assuring the quality of healthcare, and the possibility of benchmarking within general and anthroposophic healthcare. The recommendations of this study are further research of the discriminating ability of the CQ-index AH and testing of the new scales among non-AH populations in order to investigate and compare the prevalence of a broader way of practicing healthcare among other GP practices.

## Conclusion

The CQ-index AH measures patient experiences within AH validly and reliably. The quality of AH in the Netherlands is good. The CQ-index AH enables AH to take account of the quality of healthcare from the patients’ perspective systematically and in a standardized way. This is of great importance for the future perspectives of AH.

## Endnote

^a^University of applied sciences.

## Competing interests

The authors declare that they have no competing interests.

## Authors’ contributions

EK conducted the study, performed analyses and drafted the manuscript. RO performed analyses, was involved in the interpretation of findings and drafting the manuscript, RH was involved with the data collection, DD was involved in the interpretation of findings and critically revised the manuscript, EB participated in the design of the study, supervised the study and critically revised the manuscript. All authors read and approved the final version.

## Pre-publication history

The pre-publication history for this paper can be accessed here:

http://www.biomedcentral.com/1472-6963/14/148/prepub

## Supplementary Material

Additional file 1 CQIndex AH GP, CQIndex AH GP Validaded 2012.pdf, Full questionnaire. Click here for file
