# Supplementary material for: The consumer quality index anthroposophic healthcare: a construction and validation study
Source: BMC Health Serv Res. 2014 Apr 2;14:148. doi: 10.1186/1472-6963-14-148 (PMC4230405; doi:10.1186/1472-6963-14-148)
Supplement: Additional file 1 — CQIndex AH GP, CQIndex AH GP Validaded 2012.pdf, Full questionnaire. [file 1472-6963-14-148-S1.pdf]

hogeschool Leiden
lectoraat Antroposofische Gezondheidszorg

AntropoSana
onze gezondheid onze zorg

Vragenlijst

Ervaringen met de kwaliteit van de antroposofische gezondheidszorg

Consumer Quality-Index
Antroposofische Gezondheidszorg eerste lijn

Versie 4.0 december 2012

Deze vragenlijst is gebaseerd op de CQ-index huisartsenzorg overdag, aangevuld met vragen over antroposofische aspecten van de behandeling. Deze vragenlijst is ontwikkeld door het lectoraat Antroposofische Gezondheidszorg, Hogeschool Leiden en patiëntenvereniging Antroposana, in samenwerking met het beroepsveld, waaronder de Nederlandse Vereniging van Antroposofische Artsen (NVAA) en andere antroposofische beroepsverenigingen, en het CKZ en Stichting Miletus.

Code ☐☐-☐☐-☐☐☐-☐

Introductie

Deze vragenlijst heeft tot doel de kwaliteit van de eerstelijns antroposofische gezondheidszorg te meten zoals die door patiënten ervaren wordt. Zo kan de zorg beter afgestemd worden op de wensen van patiënten en kan de kwaliteit van de antroposofische zorg beter verantwoord worden naar onder andere zorgverzekeraars, overheid en politiek. De vragenlijst bestaat uit de CQI vragenlijst over de reguliere huisartsenzorg aangevuld met vragen over de antroposofische aspecten van de behandeling. Het invullen van de lijst duurt ongeveer 30 minuten.

Voordat u begint met het invullen van deze vragenlijst verzoeken wij u *de instructies op pagina 3* zorgvuldig te lezen

De vragenlijst wordt anoniem verwerkt. Dit betekent dat niemand ooit zal weten wie welke antwoorden heeft gegeven. Invullen is geheel vrijwillig en heeft *geen enkel* gevolg voor de zorg die u krijgt.
Heeft u vragen, dan kunt u bellen met XXX, telefoonnummer XXX. Ook kunt u een email sturen naar XXX

Ter extra informatie:

Wat is antroposofische geneeskunde?

Antroposofische geneeskunde is een uitbreiding op de reguliere geneeskunde. Binnen de antroposofische geneeskunde wordt er naast de reguliere mogelijkheden, gebruik gemaakt van aanvullende antroposofische diagnostiek, en antroposofische geneesmiddelen en therapieën. Binnen de antroposofische geneeskunde wordt veel aandacht geschonken aan de samenhang tussen lichaam, ziel en geest in relatie tot ziekte en gezondheid. Ook is er hierbij veel aandacht voor leefstijl, zingeving en omgevingsfactoren. Veelgenoemde en gewaardeerde kenmerken van de antroposofische geneeskunde zijn het ondersteunen en actief stimuleren van het zelfgenezend vermogen van de mens, een gelijkwaardige relatie tussen patiënt en zorgverlener, meer tijd en aandacht voor de patiënt, het ontwikkelen van natuurlijke medicijnen en een terughoudend gebruik van reguliere medicijnen. Antroposofische geneeskunde werkt even goed bij mensen die daar niets van weten.

Instructies voor het invullen van deze vragenlijst

Het is de bedoeling dat de vragenlijst wordt ingevuld door de persoon aan wie deze vragenlijst is gestuurd. Het is dus niet de bedoeling om de vragenlijst aan iemand anders door te geven. Als u te ziek bent om de vragenlijst in te vullen, hopen wij dat iemand kan helpen met het invullen van deze vragenlijst. Dit kan ook als die persoon de Nederlandse taal niet goed begrijpt. Het is de bedoeling dat de ervaring van de aangeschreven persoon wordt ingevuld.

Deze vragenlijst bestaat uit 2 delen.
Deel 1 betreft de zorg die u van uw huisarts heeft ontvangen.
Deel 2 betreft de zorg die u van een antroposofisch therapeut of behandelaar heeft ontvangen.
De allereerste vraag van deze vragenlijst is een selectievraag om u te verwijzen naar de juiste delen die voor u van toepassing zijn.
Als u zowel contact hebt gehad met uw huisarts als met een andere antroposofische behandelaar zijn beide delen van de vragenlijst voor u van toepassing. Als u beide delen invult zult u enkele dubbele vragen tegenkomen. Dit komt omdat de onderzoekers soms dezelfde informatie willen weten over de huisarts en over andere behandelaars.

Beantwoord alle vragen door een kruisje te zetten in het vakje dat links van uw antwoord staat. Sommige vragen zijn misschien niet op u van toepassing, omdat ze in het afgelopen jaar niet zijn voorgevallen. U kunt dan ‘niet van toepassing’ aankruisen. Wij willen graag weten hoe vaak of in welke mate u iets heeft ervaren. Als u in de afgelopen 12 maanden maar één keer contact heeft gehad met de huisartsenpraktijk of uw behandelaar, gaan de vragen over dat ene contact. Heeft u meerdere keren contact gehad, dan gaan de vragen over al deze keren. Indien van toepassing kunt u in plaats van *huisarts* ook *consultatief arts* lezen.
Heeft u een antwoord ingevuld, maar wilt u dat nog veranderen, zet dan het ingevulde hokje tussen haakjes en kruis een ander antwoord aan, op de volgende manier:

(☒)ja
☒ nee

1. **Met welke zorgverlener(s) heeft u de afgelopen 12 maanden contact gehad?**
   - ❑ Huisarts en/of praktijkmedewerkers binnen de huisartsenpraktijk → beantwoord alleen het gedeelte over de huisartsenpraktijk, begin bij vraag 2
   - ❑ Therapeut of andere zelfstandig behandelaar (binnen of buiten een gezondheidscentrum)→ beantwoord alleen het gedeelte over de therapeut of andere zelfstandig behandelaar, begin bij vraag 63
   - ❑ Huisarts én therapeut of zelfstandig behandelaar (binnen of buiten een gezondheidscentrum)→ beantwoord zowel het gedeelte over de huisartsen praktijk als het gedeelte over de therapeut of andere zelfstandig behandelaar, begin bij vraag 2
   - ❑ Geen van allen → dan is deze vragenlijst niet voor u bedoeld. Wilt u zo vriendelijk zijn de lijst terug te sturen?

Deel 1 Huisartsenzorg overdag

Introductie

2. **Bij welke huisarts(praktijk) bent u ingeschreven?**
   naam huisarts/praktijk

   ....

   plaats

   ....

3. **Hoe vaak heeft u in de afgelopen 12 maanden contact gehad met de huisarts(praktijk)?**
   - ❑ 1 keer
   - ❑ 2-4 keer
   - ❑ 5-9 keer
   - ❑ 10 keer of meer

4. **Heeft u bewust gekozen voor een antroposofische huisarts(praktijk)?**
   - ❑ Nee, ga door naar vraag 6
   - ❑ Ja

5. **Zo ja, wat is daarvan de reden? Meerdere antwoorden mogelijk.**
   - ❑ Advies van familie of vrienden
   - ❑ Belangstelling antroposofische gezondheidszorg
   - ❑ Toegevoegde waarde van de antroposofische gezondheidszorg
   - ❑ Elders uitbehandeld
   - ❑ Anders, nl.......................................

Huisartspraktijk

De volgende vragen gaan over de contacten (spreekuur, huisbezoek, en telefonisch contact) die u in de afgelopen 12 maanden met uw huisartspraktijk heeft gehad.

6. **Vond u de artsen en andere mede werkers van uw huisartspraktijk behulpzaam?**
   - ❑ Nooit
   - ❑ Soms
   - ❑ Meestal
   - ❑ Altijd

7. **Weet u voldoende over de organisatie van uw huisartspraktijk (spreekuurtijden, spoedtelefoon-nummers, weekenddienstregeling enz.)?**
   - ❑ Nee
   - ❑ Ja

8. **Kreeg u binnen 2 minuten iemand aan de telefoon als u overdag de huisartspraktijk belde?**
   - ❑ Nooit
   - ❑ Soms
   - ❑ Meestal
   - ❑ Altijd
   - ❑ Niet van toepassing (ik heb niet geprobeerd overdag te bellen)

9. **Als u overdag met de huisartspraktijk belde, werd u dan goed geholpen?**
   - ❑ Nooit
   - ❑ Soms
   - ❑ Meestal
   - ❑ Altijd
   - ❑ Niet van toepassing (ik heb niet geprobeerd overdag te bellen)

10. **Heeft u het als een probleem ervaren dat u eerst uw verhaal aan de assistente moest vertellen, voordat u in contact kon komen met uw huisarts?**
    - ❑ Nooit
    - ❑ Soms
    - ❑ Meestal
    - ❑ Altijd
    - ❑ Niet van toepassing (ik hoefde niets te vertellen)

11. **Beoordeelde de assistente goed dat u een afspraak nodig had met de arts?**
    - ❑ Nooit
    - ❑ Soms
    - ❑ Meestal
    - ❑ Altijd

12. **Kon u als u het nodig vond binnen 24 uur op het spreekuur terecht?**
    - ❑ Nooit
    - ❑ Soms
    - ❑ Meestal
    - ❑ Altijd
    - ❑ Niet van toepassing (heb ik nooit nodig gehad)

13. **Was de huisartspraktijk open op tijdstippen die voor u gunstig waren?**
    - ❑ Nooit
    - ❑ Soms
    - ❑ Meestal
    - ❑ Altijd

14. **Heeft u <u>te veel moeite</u> moeten doen om snel op het spreekuur terecht te kunnen?**
    - ❑ Nooit
    - ❑ Soms
    - ❑ Meestal
    - ❑ Altijd
    - ❑ Niet van toepassing (heb ik nooit geprobeerd)

15. **Was het mogelijk om op redelijke termijn een afspraak te krijgen met <u>uw eigen huisarts</u>?**
    - ❑ Nooit
    - ❑ Soms
    - ❑ Meestal
    - ❑ Altijd

16. **Behandelde de assistente u met respect?**
    - ❑ Nooit
    - ❑ Soms
    - ❑ Meestal
    - ❑ Altijd

17. **Had de assistente voldoende tijd voor u?**
    - ❑ Nooit
    - ❑ Soms
    - ❑ Meestal
    - ❑ Altijd

18. **Is uw huisartspraktijk goed toegankelijk voor kinderwagens en mensen met een lichamelijke handicap?**
    - ❑ Nee
    - ❑ Ja
    - ❑ Dat weet ik niet

19. Was de huisartspraktijk schoon?
- ❑ Nooit
- ❑ Soms
- ❑ Meestal
- ❑ Altijd
- ❑ Niet van toepassing (alleen telefonisch contact gehad)

20. Kunnen mensen in de wachtkamer horen wat er aan de balie wordt besproken?
- ❑ Nooit
- ❑ Soms
- ❑ Meestal
- ❑ Altijd
- ❑ Niet van toepassing (alleen telefonisch contact gehad)

21. Kunnen mensen in de wachtkamer horen of zien wat er in de spreek- of onderzoekskamer gebeurt?
- ❑ Nooit
- ❑ Soms
- ❑ Meestal
- ❑ Altijd
- ❑ Niet van toepassing (alleen telefonisch contact gehad)

Huisarts

De volgende vragen gaan over contacten die u <u>in de afgelopen 12 maanden</u> heeft gehad met uw huisarts (spreekuur, huisbezoek of telefonisch consult).

22. Hielp uw huisarts u binnen 15 minuten na de afgesproken tijd?
- ❑ Nooit
- ❑ Soms
- ❑ Meestal
- ❑ Altijd

23. Luisterde uw huisarts aandachtig naar u?
- ❑ Nooit
- ❑ Soms
- ❑ Meestal
- ❑ Altijd

24. Toonde uw huisarts belangstelling voor uw persoonlijke situatie?
- ❑ Nooit
- ❑ Soms
- ❑ Meestal
- ❑ Altijd

25. Nam uw huisarts u serieus?
- ❑ Nooit
- ❑ Soms
- ❑ Meestal
- ❑ Altijd

26. Gaf uw huisarts u begrijpelijke uitleg over <u>de resultaten van onderzoek</u> (bijvoorbeeld een lichamelijk onderzoek, laboratorium-onderzoek, röntgenfoto of hartfilmpje)?
- ❑ Nooit
- ❑ Soms
- ❑ Meestal
- ❑ Altijd
- ❑ Niet van toepassing (er is geen onderzoek geweest)

27. Vertelde uw huisarts u wat u wilde weten over uw klacht/gezondheidsprobleem?
- ❑ Nooit
- ❑ Soms
- ❑ Meestal
- ❑ Altijd

28. Legde uw huisarts u dingen op een <u>begrijpelijke manier</u> uit?
- ❑ Nooit
- ❑ Soms
- ❑ Meestal
- ❑ Altijd

29. Werd u door uw huisarts goed geïnformeerd over de <u>verschillende behandelingsmogelijkheden</u> voor uw gezondheidsklachten?
- ❑ Nooit
- ❑ Soms
- ❑ Meestal
- ❑ Altijd

30. Kon u meebeslissen over de behandeling of hulp die u kreeg?
- ❑ Nooit
- ❑ Soms
- ❑ Meestal
- ❑ Altijd

31. Informeerde uw huisarts u over <u>mogelijke bijwerkingen</u> van de voorgeschreven geneesmiddelen?
- ❑ Nooit
- ❑ Soms
- ❑ Meestal
- ❑ Altijd
- ❑ Niet van toepassing (ik heb geen geneesmiddelen voorgeschreven gekregen)

32. Maakte uw huisarts u duidelijk waarom het belangrijk was zijn/haar instructies/ advies op te volgen?
- ❑ Nooit
- ❑ Soms
- ❑ Meestal
- ❑ Altijd

33. Kreeg u voldoende hulp bij het 'vinden van de weg' in de gezondheidszorg (zoals informatie over behandelaars, ziekenhuizen, wachtlijsten, het maken van een afspraak en dergelijke)?
- ❑ Nooit
- ❑ Soms
- ❑ Meestal
- ❑ Altijd
- ❑ Niet van toepassing (heb ik nooit nodig gehad)

34. Werkte uw huisarts <u>goed samen</u> met uw andere zorgverleners (zoals de praktijkverpleegkundige, fysiotherapeut, thuiszorg, wijkverpleegkundige, specialist etc.)?
- ❑ Nooit
- ❑ Soms
- ❑ Meestal
- ❑ Altijd
- ❑ Niet van toepassing (ik ben niet bij een andere zorgverlener geweest)

35. Had uw huisarts aandacht voor mogelijke emotionele problemen die te maken hebben met uw gezondheid?
- ❑ Nooit
- ❑ Soms
- ❑ Meestal
- ❑ Altijd

36. Bood uw huisarts u hulp aan bij het voorkómen van ziekten of bij het verbeteren van uw gezondheid (bijvoorbeeld door controle van gewicht of bloeddruk, het geven van adviezen over voeding of leefstijl)?
- ❑ Nooit
- ❑ Soms
- ❑ Meestal
- ❑ Altijd

37. Behandelde uw huisarts u met respect?
- ❑ Nooit
- ❑ Soms
- ❑ Meestal
- ❑ Altijd

38. Had uw huisarts voldoende tijd voor u?
- ❑ Nooit
- ❑ Soms
- ❑ Meestal
- ❑ Altijd

39. Zorgde de behandeling van uw huisarts ervoor dat uw gezondheidsproblemen verminderden?
- ❑ Nooit
- ❑ Soms
- ❑ Meestal
- ❑ Altijd

40. Had u vertrouwen in de deskundigheid van uw huisarts?
- ❑ Nooit
- ❑ Soms
- ❑ Meestal
- ❑ Altijd

41. Was uw huisarts bereid met u te praten over fouten of over zaken die, naar uw mening, niet goed zijn gegaan?
- ❑ Nooit
- ❑ Soms
- ❑ Meestal
- ❑ Altijd
- ❑ Niet van toepassing (er zijn geen dingen geweest die niet goed zijn gegaan)

42. Stelde uw huisarts u op uw gemak?
- ❑ Nooit
- ❑ Soms
- ❑ Meestal
- ❑ Altijd

43. Had uw huisarts voldoende begrip voor uw klacht of aandoening?
- ❑ Nooit
- ❑ Soms
- ❑ Meestal
- ❑ Altijd

44. Gaf uw huisarts u inzicht over de achtergrond en mogelijke oorzaken van uw klacht of aandoening?
- ❑ Nooit
- ❑ Soms
- ❑ Meestal
- ❑ Altijd

45. Bood uw huisarts u de zorg die u op dat moment nodig had?
- ❑ Nooit
- ❑ Soms
- ❑ Meestal
- ❑ Altijd

46. Had uw huisarts een goede balans tussen betrokkenheid en professionele afstand?
- ❑ Nooit
- ❑ Soms
- ❑ Meestal
- ❑ Altijd

47. Heeft u de afgelopen 12 maanden in de praktijk met een andere zorgverlener dan de huisarts of assistente een afspraak gehad? ***Meerdere antwoorden mogelijk.***

- ❑ Nee,
- ❑ ja, met de (gespecialiseerd) verpleegkundige
- ❑ ja, met de diabetesverpleegkundige
- ❑ ja, met de praktijkondersteuner
- ❑ ja, met een antroposofisch therapeut (incl. verpleegkundige, diëtist, enz.)
- ❑ anders, namelijk..

Behandeling

De volgende vragen gaan over de behandeling die u van uw huisarts heeft ontvangen

48. Bent u door uw huisarts goed geïnformeerd over de antroposofische behandelingsmogelijkheden?

- ❑ Nooit
- ❑ Soms
- ❑ Meestal
- ❑ Altijd

49. Heeft uw huisarts u vrij gelaten in de keuze voor een reguliere en/of antroposofische behandeling?

- ❑ Nooit
- ❑ Soms
- ❑ Meestal
- ❑ Altijd
- ❑ Er was maar één behandelmogelijkheid
- ❑ Geen behandeling gehad

50. Hoe bent u behandeld?

- ❑ Alleen reguliere behandeling
- ❑ Alleen antroposofische behandeling
- ❑ Regulier aangevuld met antroposofische behandeling
- ❑ Geen behandeling gehad

51. Heeft de antroposofische benadering u verwachtingen gegeven ten aanzien van het verbeteren van uw gezondheidstoestand?

- ❑ Nee
- ❑ Ja
- ❑ Geen behandeling gehad
- ❑ Alleen regulier behandeld

52. Richtte uw huisarts de behandeling (ook) op het ondersteunen van de eigen herstelkrachten van uw lichaam?

- ❑ Nooit
- ❑ Soms
- ❑ Meestal
- ❑ Altijd
- ❑ Dat weet ik niet
- ❑ Geen behandeling gehad

53. Werd u door uw huisarts aangesproken in uw eigen verantwoordelijkheid voor de keuzes die u maakt met betrekking tot uw gezondheid?

- ❑ Nooit
- ❑ Soms
- ❑ Meestal
- ❑ Altijd
- ❑ Was niet aan de orde

54. Motiveerde de arts u om zelf actief aan uw gezondheidstoestand bij te dragen?

- ❑ Nooit
- ❑ Soms
- ❑ Meestal
- ❑ Altijd
- ❑ Was niet aan de orde

55. Kon u door de aanvullende benadering uw klacht of aandoening betekenis geven binnen de samenhang van uw leven en functioneren?
- ❑ Nooit
- ❑ Soms
- ❑ Meestal
- ❑ Altijd
- ❑ Was niet aan de orde
- ❑ Alleen regulier behandeld

56. Waren er bijwerkingen van de behandeling?
- ❑ Nooit
- ❑ Soms
- ❑ Meestal
- ❑ Altijd
- ❑ Geen behandeling gehad

57. Beïnvloedde de behandeling de kwaliteit van uw leven op een positieve manier?
- ❑ Nooit
- ❑ Soms
- ❑ Meestal
- ❑ Altijd
- ❑ Geen behandeling gehad

58. Kunt u aangeven waarom?
- ❑ Nee
- ❑ Ja, ik ervaar dit aan

....
....
....
....

Algemene waardering

59. Welk cijfer geeft u aan de huisartsenpraktijk? Een 0 betekent: heel erg slecht. Een 10 betekent uitstekend.
- ❑ 0 *heel erg slechte praktijk*
- ❑ 1
- ❑ 2
- ❑ 3
- ❑ 4
- ❑ 5
- ❑ 6
- ❑ 7
- ❑ 8
- ❑ 9
- ❑ 10 *uitstekende huisartspraktijk*

60. Zou u uw huisartspraktijk bij uw vrienden en familie aanbevelen?
- ❑ beslist niet
- ❑ waarschijnlijk niet
- ❑ waarschijnlijk wel
- ❑ beslist wel

61. Als u één ding zou kunnen veranderen aan de zorg die u kreeg van deze huisartsenpraktijk, wat zou dat dan zijn?

....
....
....

Over uzelf

De volgende vragen gaan over de persoon aan wie deze vragenlijst is gestuurd. Dus als u iemand helpt met het invullen van de vragenlijst, vermeld dan alstublieft de gegevens van die persoon.

101. Wat is uw leeftijd?

- ❑ 18 t/m 24 jaar
- ❑ 25 t/m 34 jaar
- ❑ 35 t/m 44 jaar
- ❑ 45 t/m 54 jaar
- ❑ 55 t/m 64 jaar
- ❑ 65 t/m 74 jaar
- ❑ 75 jaar of ouder

102. Bent u een vrouw of een man?

- ❑ vrouw
- ❑ man

103. Wat is uw hoogst voltooide opleiding? (een opleiding afgerond met diploma of voldoende getuigschrift)

- ❑ geen opleiding (lagere school niet afgemaakt)
- ❑ lager onderwijs (basisschool, speciaal basisonderwijs)
- ❑ lager of voorbereidend beroeps-onderwijs (zoals LTS, LEAO, LHNO, VMBO)
- ❑ middelbaar algemeen voortgezet onderwijs (zoals MAVO, (M)ULO, MBO-kort, VMBO-t)
- ❑ middelbaar broepsonderwijs en beroepsbegeleidend onderwijs (zoals MBO-lang, MTS, MEAO, BOL, BBL, INAS)
- ❑ hoger algemeen en voorbereidend wetenschappelijk onderwijs (zoals HAVO, VWO, Atheneum, Gymnasium, HBS, MMS)
- ❑ hoger beroepsonderwijs (zoals HBO, HTS, HEAO, HBO-V, kandidaats wetenschappelijk onderwijs)
- ❑ wetenschappelijk onderwijs (universiteit)
- ❑ anders, namelijk ………………. *(a.u.b. in blokletters)*

104. Wat is het geboorteland van uzelf?

- ❑ Nederland
- ❑ Indonesië
- ❑ Suriname
- ❑ Marokko
- ❑ Turkije
- ❑ Duitsland
- ❑ Nederlandse Antillen
- ❑ Aruba
- ❑ Anders, namelijk ....

105. Wat is het geboorteland van uw vader?

- ❑ Nederland
- ❑ Indonesië
- ❑ Suriname
- ❑ Marokko
- ❑ Turkije
- ❑ Duitsland
- ❑ Nederlandse Antillen
- ❑ Aruba
- ❑ Anders, namelijk.......................

106. Wat is het geboorteland van uw moeder?

- ❑ Nederland
- ❑ Indonesië
- ❑ Suriname
- ❑ Marokko
- ❑ Turkije
- ❑ Duitsland
- ❑ Nederlandse Antillen
- ❑ Aruba
- ❑ Anders, namelijk...........................

107. In welke taal praat u thuis het meest?
- ❑ Nederlands
- ❑ Fries
- ❑ Nederlands dialect
- ❑ Indonesisch
- ❑ (Sraran) Surinaams
- ❑ Marokkaans-Arabisch
- ❑ Turks
- ❑ Duits
- ❑ Papiamento (Nederlandse Antillen)
- ❑ Anders, namelijk..........................

108. Hoe zou u over het algemeen uw gezondheid noemen?
- ❑ slecht
- ❑ matig
- ❑ goed
- ❑ zeer goed
- ❑ uitstekend

109. Hoe zou u over het algemeen uw geestelijke/psychische gezondheid noemen?
- ❑ slecht
- ❑ matig
- ❑ goed
- ❑ zeer goed
- ❑ uitstekend

110. Heeft iemand u geholpen bij het invullen van deze vragenlijst?
- ❑ Nee, ga verder naar vraag 112
- ❑ Ja

111. Hoe heeft die persoon u geholpen?
U mag meer dan één vakje aankruisen.
- ❑ heeft de vragen voorgelezen
- ❑ heeft mijn antwoorden opgeschreven
- ❑ heeft de vragen in mijn taal vertaald
- ❑ heeft de vragen voor mij beantwoord omdat ik een kind ben
- ❑ heeft de vragen in mijn plaats beantwoord
- ❑ heeft op een andere manier geholpen, namelijk.........................
....

112. Bent u op de hoogte van de klachtenregeling (IKBC) van de antroposofische zorgaanbieders?
- ❑ Nee
- ❑ Ja

113. Wat ik verder nog wilde zeggen:..
....
....
....
....
....
....
....
....
....
....
....

Hartelijk dank voor het invullen van de vragenlijst.

Code ☐☐-☐☐-☐☐☐-☐
